# A Novel Approach to Predicting Early Pregnancy Outcomes Dynamically in a Prospective Cohort Using Repeated Ultrasound and Serum Biomarkers

**DOI:** 10.1007/s43032-023-01323-8

**Published:** 2023-08-28

**Authors:** Jesper Friis Petersen, Lennart Jan Friis-Hansen, Thue Bryndorf, Andreas Kryger Jensen, Anders Nyboe Andersen, Ellen Løkkegaard

**Affiliations:** 1 Department of Obstetrics and Gynecology, North Zealand Hospital, Hillerød, Denmark; 2 Department of Clinical Biochemistry Pharmacology, Odense University Hospital, Odense, Denmark; 3 Gynecological Clinic, 10, 3, Ny Østergade, 1101 Copenhagen, Denmark; 4 Section of Biostatistics, Institute of Public Health, University of Copenhagen, Copenhagen, Denmark; 5 Department of Clinical Research, North Zealand Hospital, Hillerød, Denmark; 6 The Fertility Clinic 4071, Copenhagen University Hospital, Rigshospitalet, Blegdamsvej 9, 2100 Copenhagen, Denmark; 7 Department of Clinical Medicine, University of Copenhagen, Copenhagen, Denmark

**Keywords:** Early pregnancy, Miscarriage, Viability, Prediction model, Estradiol, Vaginal microbiota

## Abstract

This study aimed to develop a dynamic model for predicting outcome during the first trimester of pregnancy using baseline demographic data and serially collected blood samples and transvaginal sonographies. A prospective cohort of 203 unselected women with an assumed healthy pregnancy of < 8 weeks’ gestation was followed fortnightly from 4–14 weeks’ gestation until either miscarriage or confirmed first trimester viability. The main outcome was development of a model to predict outcome from gestational age-dependent hazard ratios using both baseline and updated serial data from each visit. Secondary outcomes were descriptions of risk factors for miscarriage. The results showed that 18% of the women experienced miscarriages. A fetal heart rate detected before 8 weeks’ gestation indicated a 90% (95% CI 85–95%) chance of subsequent delivery. Maternal age (≥ 35 years), insufficient crown-rump-length (CRL) and mean gestational sac diameter (MSD) development, and presence of bleeding increased the risk of miscarriage. Serum biomarkers, including hCG, progesterone, and estradiol, were found to impact the risk of miscarriage with estradiol as the most important. The best model to predict miscarriage was a combination of maternal age, vaginal bleeding, CRL, and hCG. The second-best model was the sonography-absent model of maternal age, bleeding, hCG, and estradiol. This study suggests that combining maternal age, and evolving data from hCG, estradiol, CRL, and bleeding could be used to predict fetal outcome during the first trimester of pregnancy.

**Trial registration** ClinicalTrials.gov identifier: NCT02761772.

**Supplementary Information:**

The online version contains supplementary material available at 10.1007/s43032-023-01323-8.

## Introduction

First trimester miscarriage is the most common complication in early pregnancy. Increased maternal age is the principal risk factor [1–3] fuelled by the delayed childbearing in many societies [4]. Maternal age is also associated with increased frequency of the omen symptoms of pain and bleeding [5]. Therefore, an increasing proportion of pregnant women seek clinical reassurance of viability from an early pregnancy unit [3]. Moreover, readily available and dependable home tests for human chorionic gonadotropin (hCG) and easily accessible transvaginal sonography (TVS), have contributed to the increased incidence of reassuring visits [6]. If the initial TVS is inconclusive, current management often requires at least a two-day observation period for development of hCG and a follow-up scan [7]. Consequently, couples frequently describe severe anxiety [8, 9] and tests to ameliorate the uncertainties of first trimester viability are warranted [10]. The 2021 article series from the Lancet shed light on the significant lack of evidence surrounding miscarriage, emphasizing the need for a global reform of its care [11–13].

Early pregnancy diagnostics is usually based on demographic, biochemical or ultrasonic data. Combining all three data sources may potentially improve the prediction of outcome compared with one-parameter diagnostics. However, Pillai and colleagues [14] reviewed the performance of such combinations but found too few and too heterogenic studies in order to conduct a relevant meta-analysis. Furthermore, currently available studies considering gestational age (GA)-dependent risk factors for miscarriage are logistic regression models that do not acknowledge the possibly changing risk throughout the first trimester [15–20].

Based on the Prospective Early Pregnancy cohort of women with naturally conceived and expectedly healthy pregnancies, this study aimed to describe the best combination of variables for assessment of the dynamically altering risks of miscarriage during the first trimester.

## Methods

### The PEP Cohort

From June 2016–March 2017, the Prospective Early Pregnancy cohort (PEP cohort) recruited Danish women in early pregnancy for serial TVS and blood samples every second week until 11–14 weeks’ gestation with the primary aim of developing a biobank of data to be used in research intended to improve prediction of early pregnancy outcome. Inclusion criteria were positive urine-based hCG test and < 8 weeks’ gestation in women older than 18 years. Exclusion criteria comprised: History of recurrent pregnancy loss (≥ 3 consecutive losses), known anatomical abnormalities of the uterus or tubes, discovery of multiple gestations and pregnancies from medically assisted reproductive (MAR) treatment. At the first visit, participants were excluded if heavy vaginal bleeding, pain that prompted any surgical intervention or uterine anatomical anomalies were diagnosed. From TVS, an intrauterine pregnancy (IUP) was defined as an intrauterine yolk sac within the gestational sac or a single embryo with fetal heartbeat. If the first scan was inconclusive, the patient had a pregnancy of unknown location (PUL) and was booked for another visit per protocol. If the second scan remained inconclusive a third visit was set up. Still inconclusive at this stage, the patient was excluded from the project and referred to the local early pregnancy unit for final diagnosis and treatment. Participants contacted the study if symptoms developed between visits, and emergency consultations could be planned to ascertain viability. The woman informed the investigator if a miscarriage was diagnosed. Ectopic pregnancies were a priori decided to be excluded but did not occur. The final outcome was either a biochemical pregnancy (positive test, no pregnancy seen), anembryonic IUP or missed miscarriage (yolk sac but no embryo or embryo ≥ 6 mm without a heartbeat) or a spontaneous miscarriage (unprompted vaginal bleeding with or without conceptus remnants, preceded by IUP) [21] or confirmed viability at the TVS from 11–14 weeks’ gestation. A postpartum follow-up of predefined pregnancy and delivery data was completed in December 2018.

### Baseline Variables

Using a pre-enrollment validated digital questionnaire in an electronic case report form (SMART-TRIAL, Medei, Aalborg, Denmark), participants recorded demographic, previous medical history, lifestyle, and socioeconomic data. Participants also graded their state of physical health on a 1–5 Likert scale (5 being best). They were prompted to rate their own subjective feeling about their physical health. All male partners were asked to fulfill the same questionnaire. At the next visit, all answers were addressed for validation.

### Transvaginal Sonography and Bleeding Assessment

GA was determined from self-reported last menstrual period (LMP) as participants often were recruited before the CRL could be measured. When a CRL (in mm) was available [22] from TVS (GE Voluson i BT14, GE Healthcare, Solingen, Germany) it was recorded and used for calculations [22]. Mean gestational sac diameter (MSD) was calculated as the sum of all three orthogonal planes (in mm) divided by three. All scans were carried out by the same investigator (MD, OBGYN resident, 2 years of TVS training). The woman quantified any observed bleeding from a pictural blood assessment chart (PBAC) [23] showing the degree of stained disposable sanitary product from 0 to 4.

### Blood Samples and Laboratory Procedures

Blood was drawn using Vacutainer serum separator or K2EDTA plasma tubes (BD Diagnostics, Franklin Lakes, NJ, USA). The blood was centrifuged (Hettich Rotina 380 R, Andreas Hettich GmbH, Tuttlingen, Germany) and aliquoted to polypropylene RNase- and DNase-free microcentrifuge tubes and stored at −80 °C. The samples were analyzed eight months after the last patient had completed the trial. The PEP cohort biobank procedure have been previously published in more detail from our group in a study of pregnancy-specific reference intervals of 29 commonly used analytes [24]. All these analytes were available for this study and were used for the exploratory aim of this study; the development of early pregnancy prediction models (Supplementary Table 1).

### Statistical Analysis

Two-sample group comparisons were performed using either the Chi-squared test or t-test. For asymmetrically distributed data, the Kruskal–Wallis test was used. Baseline variables without time-dependence were modeled by logistic regression and reported as univariate odds ratios (OR) with 95% confidence intervals (CI) of miscarriage, also after multivariate adjustment according to backwards elimination of the individual variables (aOR). Time-dependent variables were visualized in scatterplots as functions of gestational age with outcome-specific mean curves obtained from generalized additive models.

The effects of the time-dependent variables on the probability of miscarriage was assessed using joint modeling of longitudinal and survival data [25]. In the joint models, the longitudinal sub-models were generalized linear mixed models with natural cubic spline parameterizations of the means and random effects. Cox proportional hazards regression with gestational age as time scale was used for the survival sub-models in which the subject-specific means from the longitudinal sub-models entered as time-dependent covariates. Dichotomized age (below or above 35 years) was also included in the Cox model as a constant covariate. The level of hCG was transformed using base-2 logarithm, and bleeding was dichotomized as bleeding or no bleeding.

Univariate (including one time-dependent covariate) and multivariate (including three time-dependent covariates) joint models were considered, and the effects of the time-dependent covariates on the probability of miscarriage were given as hazard ratios (HR). For the multivariate joint models, all possible combinations of three time-dependent covariates were analyzed giving a total of 56 models. The ability to predict miscarriage in each model was compared using WAIC (Watanabe–Akaike information criterion) [26]. This criterion provides an estimate of the out-of-sample prediction accuracy and was used to rank the models with respect to their predictive performance.

Univariate joint models were fitted using the R package *JM* [27], while multivariate joint models were fitted using the *rstanarm* package [28]. Statistical significance was set at 5%.

## Results

### Study Population

**Fig. 1:**
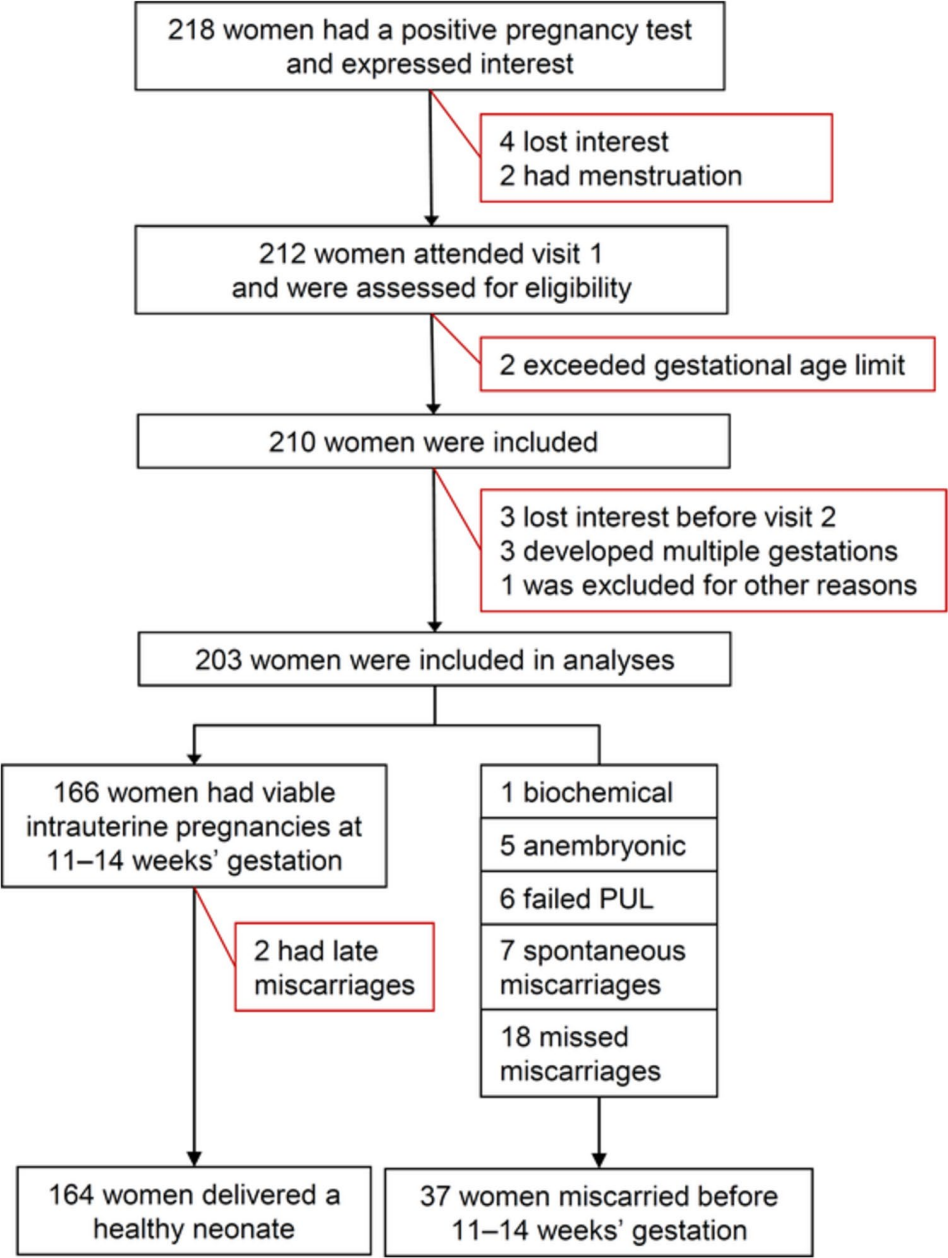
Flowchart of participants in the Prospective Early Pregnancy (PEP) cohort from expressed interest to final outcome. Red boxes show drop-outs due to various reasons from analyses

In Fig. 1 we present the flow-chart of participants in the PEP cohort from the initial expression of interest until the number of women who reached the final outcome. From 218 interested women 203 were included for analyses. Viable IUP after 11–14 weeks’ gestation was seen in 166 (82%) and 37 women (18%) miscarried. The study accumulated 715 visits (3–4 visits per participant). Two women were diagnosed with fetal chromosomal abnormalities after the first trimester and had induced abortions, they remained in the analyses as ongoing pregnancies. Live births of 164 healthy singleton neonates were recorded. In 90% (95% CI 85–95%) of women the presence of a fetal heart rate before 8 weeks’ gestation was followed by a subsequent live birth.

### Baseline Demographics and Risks

**Fig. 2:**
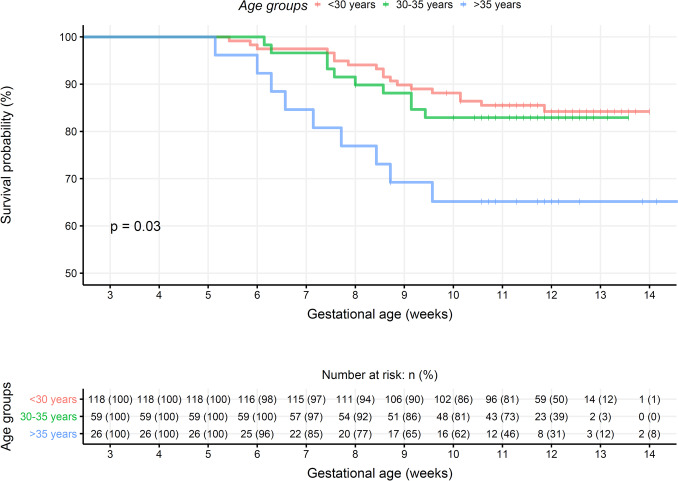
Kaplan Meier survival curve with the event of miscarriage in three maternal age groups according to gestational age. Also showing the log rank test significance for between-group comparison

Women who miscarried were on average 2 years older than women with ongoing pregnancies (31 ± 5 vs. 29 ± 4 years, *p* = 0.021). Uni- or multivariate analyses, respectively, showed that per-year maternal age increased, the risk of miscarriage was elevated by 9 or 18%, respectively (OR 1.1, 95% CI [1.0;1.2], *p* = 0.02; aOR 1.2, 95% CI [1.1; 1.3], *p* < 0.01). Table 1 shows the baseline maternal and paternal characteristics divided by outcome and Fig. 2 depicts the survival curve from Kaplan Meier statistics with the event of miscarriage according to the three age groups. Women > 35 years were namely at risk in the earliest part of the pregnancy but also showed and overall increased risk (aOR 7.5, 95% CI [2.3; 26], *p* < 0.01) while 30–35-years old had the same odds of miscarriage as younger women.

**Table 1:** Baseline maternal and paternal characteristics compared by ongoing pregnancy or miscarriage. Odds ratios (OR) of miscarriage from uni- and multivariable logistic regression

	Overall	Ongoing	Miscarriage	*p*	OR	
*n*	203	166	37		Univariable	Multivariable
Maternal data						
Age groups (years), *n (%)*				0.06		
< 30	120 (59)	102 (61)	18 (49)		–	
30–35	57 (28)	47 (28)	10 (27)		1.2 (0.5–2.8, *p* = 0.67)	1.7 (0.7–4.2, *p* = 0.26)
> 35	26 (13)	17 (10)	9 (24)		3.0 (1.1–7.7, *p* = 0.02)	7.5 (2.3–26, *p* < 0.01)
BMI (kg/m^2^), *n (%)*				0.34		
Underweight (< 18.5)	5 (3)	4 (2)	1 (3)		–	
Normal (18.5–24.9)	120 (59)	99 (60)	21 (57)		1.2 (0.1–8.5, *p* = 0.89)	1.5 (0.1–12, *p* = 0.74)
Pre-obese (25—29.9)	56 (28)	48 (29)	8 (22)		0.8 (0.3–1.8, *p* = 0.59)	0.7 (0.3–1.7, *p* = 0.40)
Obese (> 30)	22 (11)	15 (9)	7 (19)		2.2 (0.8–6.0, *p* = 0.13)	3.4 (1.1–11, *p* = 0.03)
Time to pregnancy (months), *n (%)*				0.35		
< 3	102 (50)	81 (49)	21 (57)		–	
3–6	42 (21)	35 (21)	7 (19)		0.8 (0.3–1.9, *p* = 0.59)	
6–12	20 (10)	15 (9)	5 (14)		1.3 (0.4–3.8, *p* = 0.66)	
> 12	15 (7)	15 (9)	0 (0)		NA	
Not planned	24 (12)	20 (12)	4 (11)		0.8 (0.2–2.3, *p* = 0.67)	
Gravidity, *n (%)*				0.87		
0	54 (27)	43 (26)	11 (30)		–	
1	66 (33)	54 (33)	12 (32)		0.9 (0.4–2.2, *p* = 0.76)	1.6 (0.5–5.4, *p* = 0.46)
2 +	83 (41)	69 (42)	14 (38)		0.8 (0.3–1.9, *p* = 0.60)	2.6 (0.5–11, *p* = 0.22)
Parity, *n (%)*				0.59		
0	92 (45)	73 (44)	19 (51)		–	–
1	86 (42)	71 (43)	15 (41)		0.8 (0.4–1.7, *p* = 0.59)	0.5 (0.2–1.3, *p* = 0.14)
2 +	25 (12)	22 (13)	3 (8)		0.5 (0.1–1.7, *p* = 0.33)	0.1 (0.01–0.6, *p* = 0.02)
Previous miscarriages, *n (%)*			0.46			
0	136 (67)	108 (65)	28 (76)		–	–
1	52 (26)	45 (27)	7 (19)		0.6 (0.2–1.4, *p* = 0.27)	0.5 (0.1–1.5, *p* = 0.20)
2	15 (7)	13 (8)	2 (5)		0.6 (0.1–2.3, *p* = 0.51)	0.2 (0.03–1.3, *p* = 0.12)
Regular menstrual cycle, *n (%)*				0.01		
Yes	165 (81)	136 (82)	29 (78)		–	
No	29 (14)	26 (16)	3 (8)		0.5 (0.1–1.7, *p* = 0.34)	
Unknown	9 (4)	4 (2)	5 (14)		5.9 (1.5–25, *p* = 0.01)	
Previous PID, *n (%)*	82 (40)	69 (42)	13 (35)	0.59	0.8 (0.4–1.6, *p* = 0.47)	
Previous or prevalent chronic or psychiatric disease, *n (%)*					–	
	60 (30)	50 (30)	10 (27)	0.86	0.9 (0.4–1.9, *p* = 0.71)	
Last completed education, *n (%)*				0.68		
Grad. student (1–7 years)	151 (74)	123 (74)	28 (76)		–	
Grad. student studying	25 (12)	21 (13)	4 (11)		0.8 (0.2–2.4, *p* = 0.76)	
Manual labor	22 (11)	17 (10)	5 (14)		1.3 (0.4–3.6, *p* = 0.64)	
Elementary school	5 (3)	5 (3)	0 (0)		NA	
Self-attained physical health (1–5), *n (%)*				0.19		
Level 5 (best)	40 (20)	37 (22)	3 (8)		–	
Level 4	124 (61)	97 (58)	27 (73)		3.4 (1.1–15, *p* = 0.05)	
Level 3	32 (16)	27 (16)	5 (14)		2.3 (0.5–12, *p* = 0.29)	
Level 2	7 (3)	5 (3)	2 (5)		4.9 (0.6–38, *p* = 0.12)	
Physical activity per week (hours), *n (%)*				0.52		
1–3	50 (25)	43 (26)	7 (19)		–	
4–6	75 (37)	62 (37)	13 (35)		1.3 (0.5–3.7, *p* = 0.62)	
7 +	78 (38)	61 (37)	17 (46)		1.7 (0.7–4.8, *p* = 0.27)	
Smoking, *n (%)*				0.60		
Never	117 (58)	92 (55)	25 (68)		–	
Previously	31 (15)	27 (16)	4 (11)		0.6 (0.2–1.6, *p* = 0.30)	
Stopped^a^	41 (20)	35 (21)	6 (16)		0.6 (0.2–1.6, *p* = 0.35)	
Current	14 (7)	12 (7)	2 (5)		0.6 (0.1–2.4, *p* = 0.54)	
Alcohol, *n (%)*				0.10		
None 6 months prior	66 (33)	54 (33)	12 (32)		–	
Stopped^b^	136 (67)	112 (68)	24 (65)		1.0 (0.5–2.1, *p* = 0.93)	
Current	1 (1)	0 (0)	1 (3)		NA	
Paternal data^c^						
Age groups, *n (%)*				0.01		
< 30	71 (35)	58 (35)	13 (35)		–	
30–35	72 (36)	65 (39)	7 (19)		0.5 (0.2–1.3, *p* = 0.14)	
> 35	55 (27)	35 (23)	17 (46)		2.0 (0.9–4.7, *p* = 0.10)	
Age difference				0.32		
± 5 years	151 (74)	126 (76)	25 (68)		–	
Father + 10 years	16 (8)	11 (7)	5 (14)		2.3 (0.7–7.0, *p* = 0.15)	
Father + 5–10 years	29 (14)	23 (14)	6 (16)		1.3 (0.5–3.4, *p* = 0.59)	
Mother + 5 years	2 (1)	1 (1)	1 (3)		5.0 (0.2–130, *p* = 0.26)	
Self-attained physical health (1–5), *n (%)*	0.57					
Level 5 (best)	44 (22)	39 (24)	5 (14)		–	
Level 4	98 (48)	78 (47)	20 (54)		2.0 (0.7–6.4, *p* = 0.20)	
Level 3	46 (23)	36 (22)	10 (27)		2.2 (0.7–7.5, *p* = 0.19)	
Level 2	8 (4)	6 (4)	2 (5)		2.6 (0.3–15, *p* = 0.31)	
Level 1	2 (1)	2 (1)	0 (0)		NA	
Physical activity per week (hours), *n (%)*	0.35					
1–3	54 (27)	43 (26)	11 (30)		–	
4–6	68 (34)	59 (36)	9 (24)		0.6 (0.2–1.6, *p* = 0.29)	
7 +	76 (37)	59 (36)	17 (46)		1.1 (0.5–2.7, *p* = 0.78)	
Smoking, *n (%)*				0.60		
Never	120 (59)	99 (60)	21 (57)		–	
Previously	22 (11)	16 (10)	6 (16)		1.8 (0.6–4.9, *p* = 0.29)	
Stopped^a^	11 (5)	10 (6)	1 (3)		0.5 (0.03–2.7, *p* = 0.48)	
Current	50 (25)	41 (25)	9 (24)		1.0 (0.4–2.4, *p* = 0.94)	
Alcohol, *n (%)*				0.51		
No	43 (21)	36 (22)	7 (19)		–	
Yes	155 (76)	125 (75)	30 (81)		1.2 (0.5–3.3, *p* = 0.65)	

Students t-test, chi^2^-test or Mann–Whitney *U*-test, as appropriate

*BMI* body-mass index, *PID* pelvic inflammatory disease

^a^Stopped 6 months before pregnancy

^b^Stopped at pregnancy start

^c^5 fathers declined to participate

Odds of miscarriage were significantly increased in the adjusted model for obese (> 30 kg/m^2^) women (aOR 3.4, 95% CI [1.1; 10], *p* = 0.03) and reduced for women with two or more previous deliveries (aOR 0.1, 95% CI [0.01; 0.6], *p* = 0.02) (Table 1). Unknown status of menstrual cycles was more common in the miscarriage group (14% vs. 2%, *p* = 0.008) and odds of miscarriage was increased (OR 5.9, 95% CI [1.5; 25], *p* = 0.01). Odds of miscarriage was significantly increased if a woman graded her own physical health as a 4 compared to 5 (reference group) (OR 3.4, 95% CI [1.1; 15], *p* = 0.05). The only significant paternal factor was age (*p* = 0.02). The male partner was on average three years older than the woman (33 ± 6 and 30 ± 5).

### Variables According to Gestational Age

**Fig. 3:**
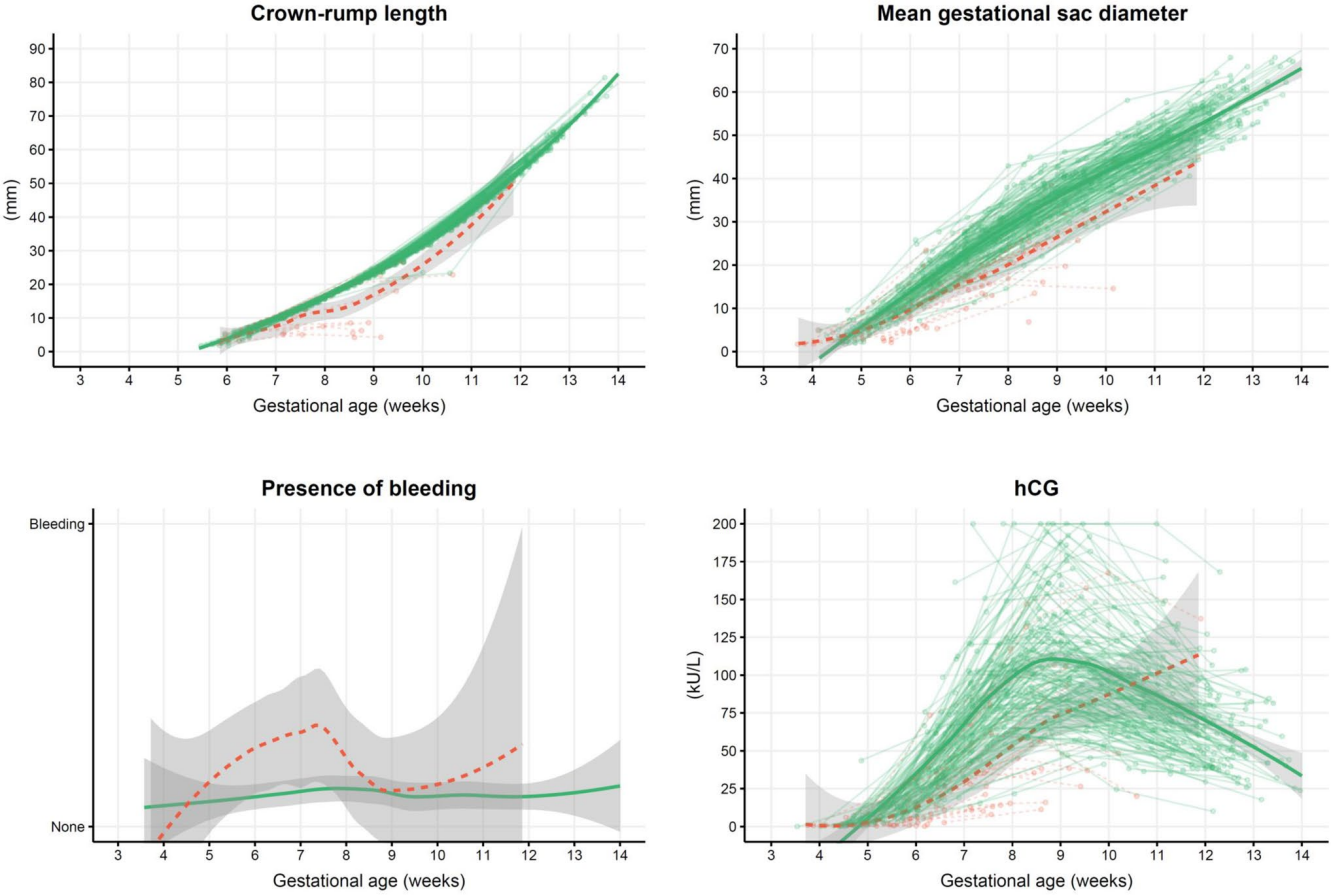

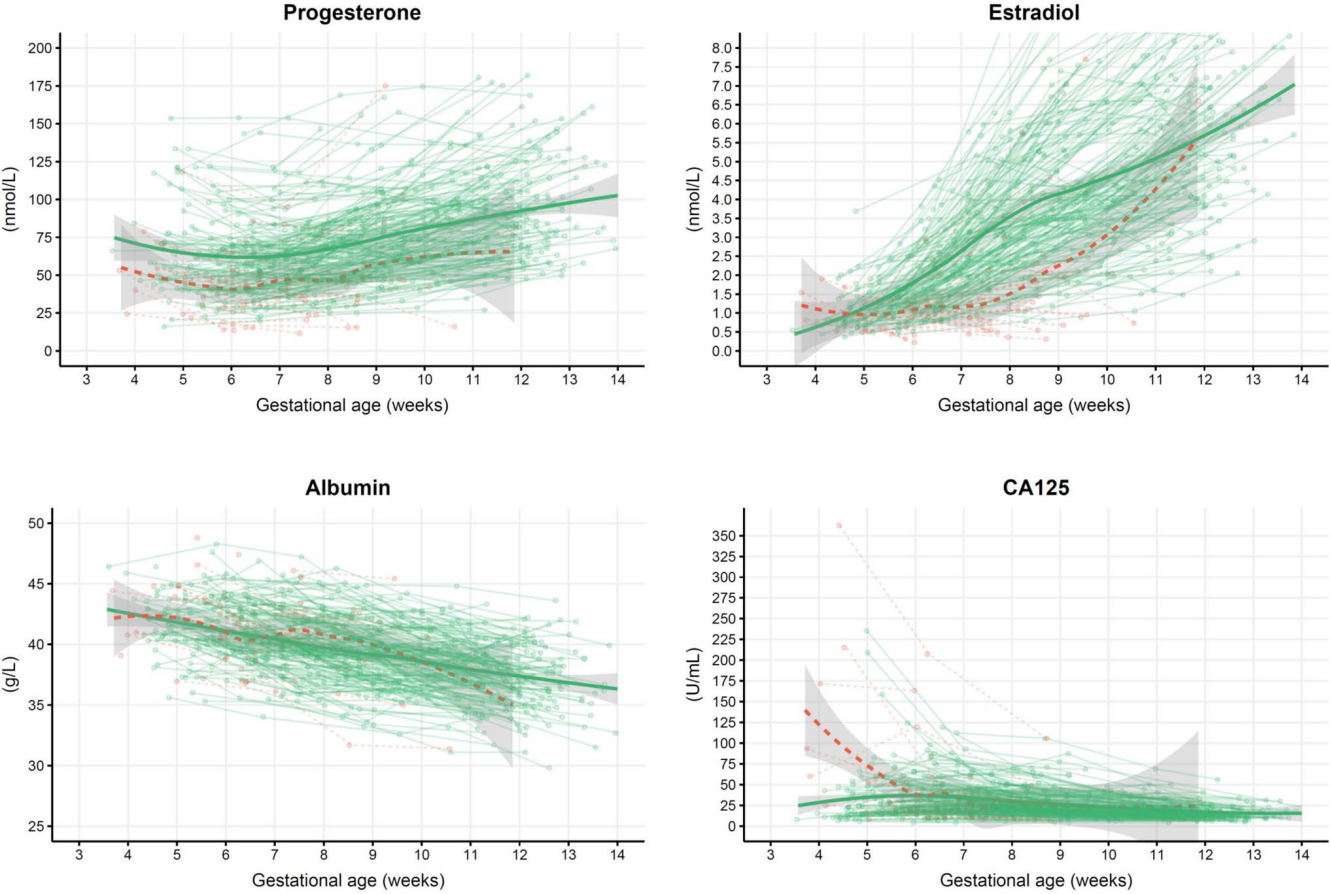
Connected observations per participant for CRL, MSD, progesterone, estradiol, albumin, CA125 and hCG colored by miscarried (red, dashed line) or ongoing (green, full line) pregnancy with a thicker-line smoothed mean and 95% CI according to gestational age

Before 7 weeks’ gestation, the CRL and MSD had similar trajectories regardless of pregnancy outcome. After this point, their trajectories differed significantly clearly visualized by the differences in the green (healthy pregnancies) and red (miscarriages) data points of Fig. 3. In Table 2 we provide a detailed evaluation of the measured levels of estradiol, progesterone and hCG in quantiles before and after 7 weeks’ gestation. The odds of a live birth typically increased comparing lower (reference) to higher quantiles both before and after 7 weeks’ gestation.

**Table 2:** Odds of a live birth by quantiles of biochemical measurands before and after 7 weeks' gestation

	N, Miscarriage / Live birth	< 7 weeks’ gestation		N, Miscarriage / Live birth	≥ 7 weeks’ gestation	
Estradiol		Quantiles (nmol/L)	OR		Quantiles (nmol/L)	OR
	16/27	0.2–1.0	ref	23/24	0.3–2.0	ref
	11/37	1.0–1.3	1.7 (0.65–4.3, *p* = 0.28)	6/41	2.0–3.0	6.5 (2.1–20, *p* < 0.05)
	5/37	1.3–2.1	4.4 (1.4–14, *p* < 0.05)	2/44	3.0–4.0	21 (4.5–99, *p* < 0.05)
	2/40	2.1–4.5	12 (2.7–52, *p* < 0.05)	2/45	4.0–11	21 (4.6–101, *p* < 0.05)
Progeste-rone		Quantiles (nmol/L)	OR		Quantiles (nmol/L)	OR
	16/27	14–40	ref	18/29	12–46	ref
	11/37	40–53	1.7 (0.66–4.2, *p* = 0.28)	8/39	46–60	3.0 (1.0–8.5, *p* < 0.05)
	4/38	53–70	5.6 (1.7–19, *p* < 0.05)	2/44	60–80	14 (3.1–60, *p* < 0.05)
	3/39	70–154	7.7 (2.0–30, *p* < 0.05)	5/42	80–169	5 (1.8–15, *p* < 0.05)
hCG		Quantiles (U/L)	OR		Quantiles (U/L)	OR
	19/27	219–7000	ref	21/26	8000–60000	ref
	6/37	7000–16000	4.8 (1.6–14, *p* < 0.05)	6/41	60,000–85000	5.5 (1.9–16, *p* < 0.05)
	7/37	16,000–37000	4.0 (1.4–11, *p* < 0.05)	2/44	85,000–115000	18 (3.8–84, *p* < 0.05)
	2/40	37,000–161000	11 (2.8–40, *p* < 0.05)	4/43	115,000–200000	8.7 (2.6–29, *p* < 0.05)

In Table 3 we provide unadjusted HRs with 95% CIs for each investigated variable from the three sources: baseline, sonography and blood. Increased MSD, CRL, estradiol, progesterone and log_2_hCG showed a significantly reduced risk of miscarriage in the serially collected GA-dependent unadjusted data (*p* < 0.001 for all mentioned). Of the remaining 26 investigated analytes: albumin, CA125, cholesterol, CRP and creatinine were also significantly associated with miscarriage. Dichotomized maternal age showed a 2.6-fold increased HR (*p* = 0.012) for miscarriage in the ≥ 35 years old group. Bleeding was also dichotomized as present or absent with a 1.1-fold increased miscarriage HR (*p* = 0.008) if present.

**Table 3:** Unadjusted hazard ratio (HR) with 95% CI for miscarriage in all statistically significant variables. Categorical variables (*italics*) show the presence effect. Continuous variables show the effect of a one-unit (or otherwise specified) increase (CRL: crown-rump-length, MSD: mean gestational sac diameter, CA: cancer antigen, hCG: human chorionic gonadotropin)

		95% CI		*p*
	HR	Lower	Upper	
*Age (*≥ *35 years)*	*2,6*	*1,2*	*5,6*	*0,012*
*Bleeding (present)*	*1,1*	*1,0*	*1,3*	*0,008*
CRL	0,76	0,69	0,82	< 0.001
MSD	0,72	0,64	0,79	< 0.001
Albumin	1,3	1,0	1,5	0,013
CA125	0,62	0,44	0,89	0,010
Cholesterol	1,5	1,2	1,9	0,002
C-reactive protein	1,1	1,0	1,2	0,004
Creatinine	1,1	1,0	1,2	0,010
Estradiol (+ *0.1 nmol/L*)	0,80	0,75	0,84	< 0.0001
Progesterone *(*+ *5 nmol/L)*	0,76	0,74	0,77	< 0.0001
hCG (log_2_)	0,36	0,26	0,48	< 0.001

Figure 3 shows all eight selected variables for modelling, colored by outcome and plotted according to GA with lines between the values for each woman.

### Combined Biomarkers

From the unadjusted HR in Table 3, the following factors were selected for the modelling of dynamic survival probability, combining no more than three variables: CRL, MSD, bleeding, estradiol, progesterone, albumin, CA125 and hCG. Dichotomized age was added to all models for a total of 56 combinations. The results of each model are available in Supplementary Table 2 and ranked from 1–56 by decreasing prediction accuracy (increasing values of WAIC). From all models, the best combination of variables for prediction of miscarriage was age, hCG, CRL and bleeding. The second-best model was the sonography-absent model of maternal age, bleeding, hCG, and estradiol.

**Fig. 4:**
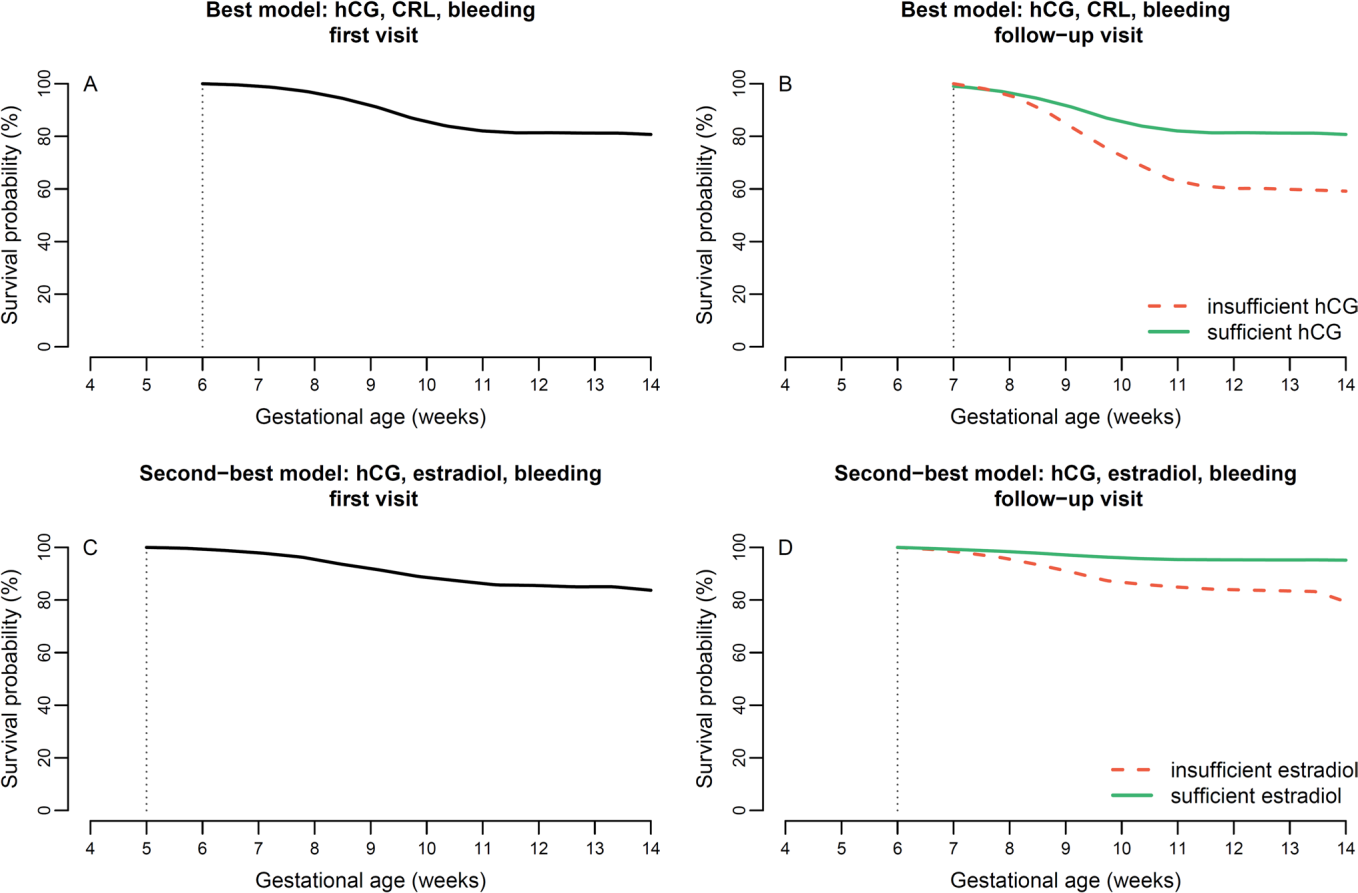
Pregnancy survival probability curve for a theoretical 36-year old woman who presents in week 6 with vaginal bleeding, CRL of 5 mm and hCG of 15.000 U/L (**A**). One week later (**B**) the CRL sufficiently increased at 8 mm but the probability of survival is shown according to two types of hCG increase (sufficiently: full line at 30.000 U/L vs. insufficiently: dashed at 16.000 U/L). The second-best combination of variables predicted the outcome for the same woman at her first visit (week 5, estradiol at 1.0 nmol/L) (**C**) and one week later (**D**) according to different estradiol increases (full line, 2.0 nmol/L vs. dashed, 1.1 nmol/L)

In Fig. 4 (see the figure legend for all details) we provide a theoretical example of survival probability calculations from updated values between the first and a follow-up visit using both the best model of hCG, CRL and bleeding (panel A and B) (showing the change in survival probability from an insufficient hCG development) and the second-best model (panel C and D) combining bleeding, hCG and estradiol (showing the change in survival probability from an insufficient estradiol development).

## Discussion

Our study used a longitudinal dataset from the first trimester and introduced a novel approach to predicting outcomes using joint statistical modeling. The longitudinal data highlighted key differences between the investigated variables according to GA. We found that a combination of maternal age, bleeding, hCG, and CRL was the most accurate predictor of outcome. When CRL was unavailable, estradiol provided a useful alternative.

Interestingly, our study found that estradiol was a more effective predictor of outcome than progesterone, which was unexpected based on previous research [3, 19, 20]. Although the ideal tool for predicting miscarriage has yet to be developed, it has been the subject of many investigations. Previous studies have primarily focused on progesterone as the main biochemical predictor of miscarriage. However, in our data, the best combined model including progesterone was ranked 20th (with CRL and hCG). By examining women throughout the first trimester, we found that the dynamic alterations of hCG and estradiol provided a more accurate description of miscarriage prediction. Whittaker et al. conducted a prospective cohort study from 1978–1985, which resembled our design, to describe the first trimester gestational trajectory of serially collected estradiol, progesterone, and hCG related to miscarriage [29]. Their findings corroborate ours, indicating that dynamic changes in estradiol and progesterone are important in identifying impending miscarriages before 7 weeks of gestation. Moreover, a 2016 meta-analysis also supports the potential utility of estradiol in predicting miscarriage, and a recent retrospective study identified estradiol as the most effective marker of miscarriage prediction [7, 30]. In a recent ESHRE abstract, a plausible explanation of serum estradiol and outcome prediction was proposed. The study collected paired vaginal microbiome and serum estradiol (and progesterone) samples from 100 women during the first trimester. It was found that women who had a live birth despite having low levels of pregnancy-favorable Lactobacillus species [31] had significantly higher levels of estradiol compared to those with similar favorable Lactobacillus species depletion who miscarried. This was not the case for progesterone. Additionally, longitudinal samples from these high-estradiol women who had a live birth showed increasing levels of Lactobacillus species later in pregnancy [32]. Estradiol has been shown to increase glycogen storage in the vaginal mucosal lining, hypothetically supporting the dominance of Lactobacillus species [31].

Considering sonographic markers of viability, Bottomley et al. developed a prediction model for women with an intrauterine pregnancy of uncertain viability without blood sampling. Combining maternal age, bleeding assessment, GA, MSD and presence of a yolk sac provided an AUC of 0.77 [18]. The authors re-tested the original models and updated with a new GA-independent version in an external prospective validation study and confirmed the results at an AUC of 0.85 [16]. Finally, the group used the model in the original cohort of all consecutively examined women (irrespective of first TVS findings and therefore resembling our population), and got an AUC of 0.92 in 1435 participants [17]. Although these studies demonstrate accurate prediction of viability with non-invasive methods, they are subject to potential inaccuracies due to patient- and operator-dependent factors such as bleeding quantification and TVS quality. Moreover, their estimates were based on logistic regression models using only one or two values from each participant, which may introduce selection bias. In contrast, our study utilized individualized time-varying hazard ratios, providing a more precise assessment of risk.

The coveted ideal prediction model for fetal viability would likely utilize the combination of multiple data sources. In 2003, Elson et al. developed a logistic regression-based model including serum progesterone, MSD and maternal age in women with an intrauterine pregnancy of uncertain viability. The area under the receiver operating characteristics (ROC) curve (AUC) was 0.97 [20] and a follow-up trial found an AUC of 0.85 [19]. In the latter trial, testing was more likely to be carried out in women with an expected higher risk of miscarriage that may explain the lowered performance. Also, still at an experimental stage, the test was only used in about 9% of eligible patients adding to the selection bias.

The recruitment of patients in the PEP cohort before onset of symptoms allowed us to evaluate risk factors for miscarriage prior to the entry of care [33]. Previously well-described, we found an increased risk of miscarriage by maternal age ≥ 35 years [1, 3]. This may be explained by increased aneuploidy in older women and these losses also occurred more frequently before 7 weeks’ gestation (Fig. 2) [34]. Obesity (BMI > 30 kg/m^2^) increased and birth of two or more children decreased the risk of miscarriage [35, 36], respectively. Ideally, our study setup and power would have allowed for an evaluation of the effect modification of BMI on all serially collected variables which was not possible from the available sample size. A 2014 systematic review clearly documented association between smoking and miscarriage [37]. Probably due to a lack of statistical power, our study found no association between smoking status and risk of miscarriage. Unknown menstrual cycle status increased the risk of miscarriage but with wide confidence limits. The nine women who gave the answer all became pregnant shortly after giving birth or finishing a long use of hormonal contraception. When asked about usual menstrual pattern, one woman reported irregular periods. Backwards elimination removed this variable from the adjusted calculations. Collectively, we consider this a chance finding of minor importance.

The risk of miscarriage increased with lower physical well-being scores. More women at baseline (before outcome was known) in the group with a viable fetus reported perfect physical satisfaction (grade 5). The motivation for filling in lower grades were not further explored but may be linked to unhealthy behavior known to increase the risk of miscarriage (like smoking or being overweight). Further, older women with the knowledge about increased risk of miscarriage with age may apply this concern when having to describe their won health. This variable was also of minor statistical importance and excluded from the adjusted calculations. This finding is consistent with Maconochie et al.’s retrospective analysis of more than 6,000 women’s latest pregnancy, which found a lower risk of miscarriage in those who felt “well enough to fly or have sex.” [36]. No paternal variables were significant.

Our data only allowed a combination of three variables (besides maternal age) for estimation of dynamically altering risk. From the statistically significant covariates, we selected the feasible variables of CRL, MSD, bleeding (dichotomized), estradiol, progesterone and hCG for modelling. We added CA-125 as a novel candidate that have previously shown discriminative potential in a 2001 study by Schmidt et al. and found to be the best marker of threatening miscarriage in a 2016 Human Reproduction Update paper. Our study did not corroborate this finding. Albumin is known to decrease significantly in a healthy first trimester pregnancy as data from the PEP cohort has shown previously. It was therefore added to explore a possibility for differentiation that was not found [7, 38–41]. In 2019, a prospective study found that dichotomized bleeding was equal to grading the amount of first trimester bleeding in prediction of later gestational pathology [42]. The unreliable estimation of bleeding was therefore removed, reducing patient-subjective recall bias. Given the fact that heavy bleeding is a detrimental risk factor of miscarriage we would have included the graded data if statistically possible [18].

Fetal ploidy was unknown as product of conception tests was unfeasible. Moreover, our aim was to develop a model that could alleviate some of the anxiety of early pregnancy and the currently available diagnostic work-up does not distinguish by ploidy. Individualized genetic counselling may change this in the future [43, 44] alongside technological advancements in the artificial intelligence interpretation of early pregnancy ultrasonography [45]. Fetal heart rate (FHR) quantification has been shown to be a useful predictor of viability [14] but was left out for safety reasons as the heat-induction from doppler-based estimation of FHR may affect developing embryos before 10 weeks’ gestation according to the manufacturer.

The overall sample size was insufficient for an estimate of each model’s ability to predict positive and negative cases (e.g. AUC). This unfortunate limitation was primarily due to the challenging logistics of serial collection but acknowledge our aim of evaluating potential models. Any follow-up trials for validation of the models should take great care to get the sample size appropriate.

Developing tools for prediction of outcome comes at the price of possibly being able to predict increased risk of miscarriage in an otherwise low-risk pregnancy providing more anxiety contrary to the intention of development. Implementation of such models should therefore focus on women with a clear indication for testing, such as previous miscarriages or maternal age > 35 years.

In our prospective cohort of naturally conceived, serially followed and expectedly healthy pregnancies, maternal age, bleeding, hCG, and CRL provided the best model. A TVS-independent model of maternal age, bleeding, hCG, and estradiol also performed well. Surprisingly, estradiol was a better predictor of miscarriage than progesterone.

### Supplementary Information

Below is the link to the electronic supplementary material.

Supplementary file1 (XLSX 13 KB)

Supplementary file2 (XLSX 28 KB)

## Data Availability

The authors commit to making the relevant anonymised patient level data available on reasonable request.

## Declarations

### Transparency Declaration

The corresponding author affirms that this manuscript is an honest, accurate, and transparent account of the study being reported; that no important aspects of the study have been omitted; and that any discrepancies from the study as planned (and, if relevant, registered) have been explained.
